# Assessing Insulin Sensitivity and Postprandial Triglyceridemic Response Phenotypes With a Mixed Macronutrient Tolerance Test

**DOI:** 10.3389/fnut.2022.877696

**Published:** 2022-05-11

**Authors:** John W. Newman, Sridevi Krishnan, Kamil Borkowski, Sean H. Adams, Charles B. Stephensen, Nancy L. Keim

**Affiliations:** ^1^Western Human Nutrition Research Center, Agricultural Research Service, USDA, Davis, CA, United States; ^2^Department of Nutrition, University of California, Davis, Davis, CA, United States; ^3^West Coast Metabolomics Center, Genome Center, University of California, Davis, Davis, CA, United States; ^4^Department of Surgery, Davis School of Medicine, University of California, Davis, Sacramento, CA, United States; ^5^Center for Alimentary and Metabolic Science, Davis School of Medicine, University of California, Davis, Sacramento, CA, United States

**Keywords:** insulin sensitivity, meal challenge test, phenotyping, triglyceridemia, postprandial triglyceridemia, fat tolerance test, insulin patterns

## Abstract

The use of meal challenge tests to assess postprandial responses in carbohydrate and fat metabolism is well established in clinical nutrition research. However, challenge meal compositions and protocols remain a variable. Here, we validated a mixed macronutrient tolerance test (MMTT), containing 56-g palm oil, 59-g sucrose, and 26-g egg white protein for the parallel determination of insulin sensitivity and postprandial triglyceridemia in clinically healthy subjects. The MMTT was administered in two study populations. In one, women with overweight/obese BMIs (*n* = 43) involved in an 8-week dietary intervention were administered oral glucose tolerance tests (OGTTs) and MMTTs within 2 days of each other after 0, 2, and 8 weeks of the dietary intervention. In the other, 340 men and women between 18 and 64 years of age, with BMI from 18–40 kg/m^2^, completed the MMTT as part of a broad nutritional phenotyping effort. Postprandial blood collected at 0, 0.5, 3, and 6 h was used to measure glucose, insulin, and clinical lipid panels. The MMTT postprandial insulin-dependent glucose disposal was evaluated by using the Matsuda Index algorithm and the 0- and 3 h blood insulin and glucose measures. The resulting MMTT insulin sensitivity index (ISI_MMTT_) was strongly correlated (*r* = 0.77, *p* < 0.001) with the OGTT-dependent 2 h composite Matsuda index (ISI_Composite_), being related by the following equation: Log (ISI_Composite_) = [0.8751 x Log(ISI_MMTT_)] –0.2115. An area under the triglyceride excursion curve >11.15 mg/mL h^–1^ calculated from the 0, 3, and 6 h blood draws established mild-to-moderate triglyceridemia in agreement with ∼20% greater prevalence of hypertriglyceridemia than fasting indications. We also demonstrated that the product of the 0 to 3 h and 3 to 6 h triglyceride rate of change as a function of the triglyceride incremental area under the curve optimally stratified subjects by postprandial response patterns. Notably, ∼2% of the population showed minimal triglyceride appearance by 6 h, while ∼25% had increasing triglycerides through 6 h. Ultimately, using three blood draws, the MMTT allowed for the simultaneous determination of insulin sensitivity and postprandial triglyceridemia in individuals without clinically diagnosed disease.

**Clinical Trial Registration:**

[https://clinicaltrials.gov/], identifier [NCT02298725; NCT02367287].

## Introduction

Insulin sensitivity and plasma triglyceride levels are important factors in the evaluation of cardiometabolic risk, and various approaches to their assessment are available. In type 2 diabetes and its pre-clinical manifestations, a loss of insulin sensitivity leads to an exaggerated surge of blood insulin and glucose, following carbohydrate intake (1). In some individuals, insulin resistance is accompanied by hypertriglyceridemia, an independent cardiometabolic risk factor (2). While fasting triglycerides (TGs) have been classically used to establish triglyceridemic status, postprandial hypertriglyceridemia appears to provide a better predictor of cardiovascular disease risk in those without frank (i.e., pre-clinical) diabetes (2). Moreover, TGs measured 2 to 4 h postprandially, unlike fasting, have strong associations with cardiovascular events independent of both insulin resistance (IR) and high-density lipoprotein cholesterol (HDL-c) levels (3). Therefore, the simultaneous assessment of insulin sensitivity and postprandial triglyceridemic responses has value to clinical cardiovascular risk management and research, exploring the interindividual variability in this metabolic phenotype.

Approaches to assessing insulin sensitivity have understandably been glucose centric, with clinical indices based on either the homeostatic balance of insulin and glucose or how an individual regulates insulin in response to a standardized 75-g glucose challenge (4). While the glucose to insulin ratio provides potentially useful indication of insulin sensitivity in the absence of diabetes, this measure loses utility with elevated fasting glucose (5). Clinically relevant models validated against the gold standard euglycemic clamp include those assessing basal glucose and insulin homeostasis [e.g., the homeostasis model of insulin resistance (HOMA-IR) and quantitative insulin sensitivity check index (QUICKI)], and those incorporating the postprandial response to a glucose challenge [e.g., Matsuda insulin sensitivity index (ISI_Matsuda_), beta-cell disposition index], probing a glucose challenge response provides opportunities to assess pancreatic function and peripheral glucose disposal (6, 7). However, mixed macronutrient tolerance tests (MMTTs) are gaining popularity as they allow for a broader probe of the nutritional phenotype, including evaluations of metabolic flexibility (i.e., fuel switching), insulin sensitivity, and lipid tolerance (8–14).

Studying participants with and without type 2 diabetes, a standardized liquid MMTT was previously shown to effectively interrogate multiple metabolic parameters, including postprandial blood insulin, glucose, TGs, adipose lipolysis, amino acid metabolism, and more (15). In the current study, we characterized a similar MMTT but replacing a dairy-based protein powder with egg whites and dextrose with sucrose, reporting here its use for the assessments of surrogate measures of insulin sensitivity and lipid tolerance simultaneously in a non-diabetic population. We evaluated a liquid MMTT of 56-g palm oil, 59-g sucrose, and 26-g egg white protein to perturb both insulin and TG homeostasis. Ultimately, we show: 1) that the developed protocols provide robust measures of insulin sensitivity and postprandial triglyceridemia; 2) that the magnitude of the triglyceridemic response is variable across the population; 3) that postprandial triglycerides increased for up to 6 h in a large segment of this generally healthy study population; and 4) that ∼2% of the population shows a minimal postprandial triglyceride increases in this time frame.

## Materials and Methods

### Study Participants

**FIGURE 1:**
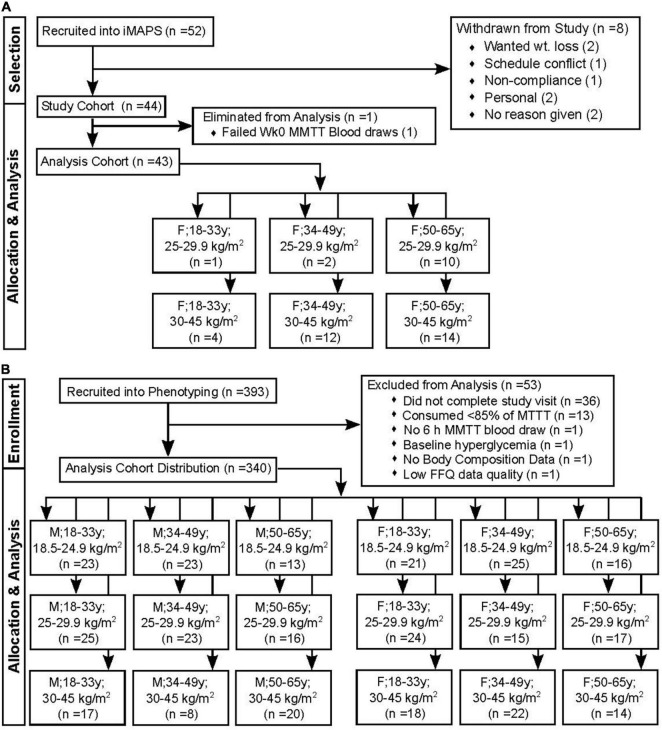
Study recruitment and data restrictions for the mixed macronutrient tolerance test (MMTT) analysis. **(A)** The individual Metabolism and Physiological Signatures Study (iMAPS) recruited 52 female (F) individuals with BMIs in the overweight to the obese range. Of these, 44 completed the entire 8-week intervention. A single individual was lost to the triglyceride analysis due to difficulty in blood collection **(B)** The WHNRC nutritional phenotyping study recruited 393 male (M) and F participants with attempts to balance into 3 age and BMI ranges. Of these, 340 were retained for analyses.

Participants were from two independent clinical studies conducted at the United States Department of Agriculture - Agricultural Research Service - Western Human Nutrition Research Center (WHNRC) in Davis California. The individual Metabolism and Physiological Signatures Study (iMAPS; *ClinicalTrials.gov:* NCT02298725) recruited pre- and postmenopausal women with overweight to obese BMIs, who had <150 min/week of physical activity and ≥1 cardiometabolic risk factor (*n* = 44) in an 8-week feeding intervention to test the impact of diets, meeting the Dietary Guidelines for Americans on cardiometabolic risk factors. Inclusion criteria included age 20–65 years, BMI of 25–39.9 kg/m^2^, resting blood pressure of ≤140/90 mm Hg, and/or evidence of impaired glucose homeostasis and/or elevated fasting TGs as previously described (16). Subject body composition was determined at 0 and 8 weeks by dual-energy X-ray absorptiometry (DXA; Hologic Discovery QDR Series 84994; Hologic, Inc.). The participants were screened to ensure a sedentary lifestyle (i.e., <150 min of exercise per week) and asked to maintain their normal physical activity levels during the 8-week intervention. To confirm adherence, activity was monitored for 7-day periods 4 times over the course of the 8-week study using waist-worn accelerometers (Respironics^®^ Actical™; Philips North America Co, Cambridge MA). The WHNRC Cross-Sectional Nutritional Phenotyping Study (Phenotyping Study; *ClinicalTrials.gov:* NCT02367287) recruited a cohort of 393 generally healthy individuals living near Davis, CA, starting in May of 2015. Population demographics were similar to the 2010 CA census: 61.1% (versus 60.6%), White; 15.4% (versus 13.7%), other race; 12.3% (versus 14.1%), Asian; 4.8% (versus 5.8%), Black; 4.8% (versus 4.7%), two or more races; 0.6% (versus 0.8%), native American; 1.1% (versus 0.4%), native Hawaiian or Pacific Islander. The study was designed to probe sex by age by BMI differences. Therefore, recruitment included both males and females between the ages of 18 and 65 and with BMI of 18.5–40 kg/m^2^ (17), and enrollment efforts strove for equal distributions across the three ages and three BMI bins by sex, and, therefore, do not constitute a true cross-section of the population. Body composition was determined by DXA and the participants underwent a variety of physiological and psychological tests to be presented in other reports. Physical activity was monitored between the two study visit days using waist-worn accelerometers (Respironics^®^ Actical™; Philips North America Co). The final cohorts available for analysis and their sex-x-age-x-BMI-class distributions are presented in Figure 1.

### Clinical Chemistry

Plasma glucose was measured by an enzyme-linked colorimetric assay on a Clinical Chemistry Analyzer (Cobas Integra 400 +; Roche Diagnostics Corporation). Serum insulin concentrations were measured by a competitive binding assay on an automatic analyzer (Cobas E 411; Roche Diagnostics). In the phenotyping study, TGs and cholesterol were measured on a Cobas Integra 400 +, while, in iMAPS, plasma TGs and cholesterol were run on an automatic analyzer (Beckman Coulter DXC800) at the UC Davis Health, Department of Pathology and Laboratory Medicine Clinical Laboratory. All assays met manufacturers’ recommendations with inter- and intraday variability of <2%.

### Oral Glucose Tolerance Test

At weeks 0, 2, and 8 of iMAPS, 75 g of glucose was administered orally to volunteers after a ∼12 h overnight fast. Blood was collected by an antecubital vein indwelling catheter within 5 min of glucose ingestion, with four subsequent blood samples collected at 0.5, 1, 1.5, and 2 h as previously described (16).

### Mixed Macronutrient Tolerance Test Pre-test Meals

All the participants had consumed a study-specific standardized pretest dinner the night before the MMTT. In iMAPS, the pretest dinner consisted of a chicken, cheese and bean burrito, corn chips, and lemonade, containing 34-g fat, 123 g of carbohydrates, and 32 g of protein (926 kcal). The fat consisted of ∼ 1:2:1 SFA/MUFA/PUFA, while the carbohydrates contained ∼25 g of simple sugars, 80 g of starch, and 20 g of fibers. In phenotyping, the pretest dinner consisted of stir-fried rice with vegetables, egg, and sweet and sour sauce, accompanied by roasted potato soup, a lemonade drink and a sorbet dessert. The meal contained ∼17-g fat, ∼160-g carbohydrates, and ∼20 g of protein (873 kcal). The fat consisted of ∼ 1:2:1 SFA/MUFA/PUFA, while the carbohydrates contained ∼90 g of simple sugars, 30 g of starch, and 9 g of fibers. Detailed compositions and links to meal recipes can be found in Supplemental Information to this manuscript.

### Mixed Macronutrient Tolerance Test

The WHNRC MMTT was patterned after a similar meal developed as the “PhenFlex” challenge (15). The WHNRC MMTT contains palm oil, sucrose, and pasteurized liquid egg white as the main ingredients, with xanthan gum, gum celluloses, and maltodextrin as emulsifying agents, and vanilla, almond, and artificial butter flavorings to improve palatability. See Table 1 for the challenge meal recipe. We replaced the PhenFlex challenge dextrose with sucrose to better reflect the types of simple sugar regularly ingested, and to allow the potential impact of sucrose-derived fructose on lipid metabolism. The inclusion of a protein source also better reflects a standard meal, allows for protein-fat-carbohydrate interactions and provides an opportunity to evaluate variance in postprandial protein metabolism (15). The latter data have been collected as part of a metabolomics effort and will be the focus of a future manuscript. In iMAPS, the participants received the MMTT within 2 days, following the OGTT. In the phenotyping study, participant anthropometry, body composition, and physiological assessment data were collected; training for at-home dietary data collection and accelerometer placement and pre-test meals described below were provided on their 1st-study test day visit (17). The MMTT was conducted on the phenotyping 2nd-study visit test day.

**TABLE 1:** A liquid-mixed macronutrient tolerance test recipe.

Ingredient	Source	Amt (g)	Wt %
pasteurized liquid egg whites	Lucerne Foods, Inc.	2106	70%
Organic palm oil shortening	Spectrum Organic Products, LLC	456.5	15%
Granulated white sugar	C&H; ASR Group	421.4	14%
Cellulose gum thickener	Thik& Clear; NUTRA-*Balance*	8.30	0.3%
Xanthan gum	Bob’s Red Mill Natural Foods	4.70	0.2%
Pure vanilla extract	McCormick& Company, Inc.	1.00	0.03%
Almond extract	McCormick& Company, Inc.	1.00	0.03%
Imitation butter flavor	McCormick& Company, Inc.	1.00	0.03%

*To prepare 7 individual portions, listed ingredients are combined and homogenized in a food grade blender by two rounds of three low-speed pulses and 30 s of low-speed blending, followed by 60 s of high-speed blending. Portions (400 ml) are transferred to 500-ml Nalgene bottles, labeled with the preparation date and stored at 20°C for <6 mo.*

Each participant was served the MMTT formula and 60 ml of water to cleanse the pallet after MMTT ingestion. A fasting blood sample was collected, the MMTT was consumed within 5 min, and postprandial blood collections occurred at 0.5, 3, and 6 h. Additional water intake was restricted until after the 0.5 h postprandial blood draw, followed by *ad libitum* deionized water for the remainder of the day. The first study participant was provided 350 g of the MMTT; however, material loss due to viscosity and sticking to the drinking vessel was noted, and so all other participants received 403 g to account for this (∼12 fluid oz. or 340 ml). The 403-g MMTT portion allowed for consumption of 380 ± 22 g (∼95%) of the provided material, controlling for the mixture viscosity. In the phenotyping study, of 357 normoglycemic participants completing the meal challenge test, 17 were excluded from the analysis as described in Figure 1. Thirteen of these consumed <85% of the 380-g average dose, with two <65%. The retained phenotyping (*n* = 340) and iMAPS participants consumed 99 ± 3% and 102 ± 4 of the average doses, respectively. For the participants used in the subsequent analysis, this equated to a delivered dose of 56 ± 2 g of palm oil, 59 ± 2 g of sucrose, and 26 ± 1 g of egg white protein. Based on compositional analyses performed by Covance Laboratories (Madison, WI), a dose of 380 g would provide an 840 kcal caloric load (fat– 60 cal%; carbohydrate – 28 cal%, protein– 12 cal%; moisture – 62.5 %). The fatty acid composition by weight of the total fat was 43% palmitate (16:0), 40% oleate (C18: 1n9), 9% linoleate (18:2n6), 4% stearate (18:0), and <1 % other detected fatty acid residues.

### Indirect Calorimetry

The metabolic rate was assessed by trained physiologists using indirect calorimetry. Automated metabolic carts (TrueOne 2400, ParvoMedics) were used to measure resting and postprandial metabolic rates using an open circuit system. Measurement times closely coincided with blood collection times: 0, 0.75–1, 3, and 6 h postprandial. The participants had rested quietly for 5–10 min before beginning the assessment, and data were collected for ∼15–20 min with the participants in a semi-reclined position, wearing a securely fitting facemask, covering the nose and the mouth. Participant exhalation of inhaled room air was directed to the metabolic cart mixing chamber for volume and gas analyses. Respiratory exchange ratios (RERs) are calculated using observed volume of oxygen (V̇O_2_) consumed and volume of carbon dioxide (V̇CO_2_) produced using the equation V̇CO_2_/V̇O_2_. The resting and postprandial energy expenditure (EE) were estimated without urinary nitrogen correction using the Weir equation: EE = [(3.94 x V̇O_2_) + (1.1 x V̇CO_2_)] (18).

### Insulin Sensitivity

Among the various reported procedures for estimating insulin sensitivity using plasma measures of glucose and insulin, the Matsuda index has wide acceptance and the strongest correlation with the euglycemic-hyperinsulinemic clamp (19). Insulin sensitivity was estimated from the fasting and postprandial insulin-glucose relationship as proposed by DeFronzo and Matsuda [DeFronzo and Matsuda, (20)]. Specifically, the 0 and 2 h glucose and insulin data from the OGTT were used to calculate the “composite” insulin sensitivity index (ISI_Composite_) (20, 21). An MMTT insulin sensitivity index (ISI_MMTT_) was calculated in a parallel fashion using the 0 and 3 h glucose concentration (mg/dL) and insulin concentrations (mU/L) with the following equation:

ISIMMTT=10,000/(√[Glucx0hInsulinx0hGlucx3hInsulin)3h]

Importantly, a 2 h blood draw was not performed during the MMTT, and the 3 h blood draw represents a compromise, allowing the assessment of both insulin sensitivity and triglyceridemia while minimizing blood draws. For purposes of comparing the OGTT and MMTT, the participants were considered insulin resistant (IR) if the ISI_Composite_ was <4.3, and insulin sensitive (IS) when above this cut-off (4, 22–24). It should be recognized that this ISI cut-off is not universally recognized and is somewhat arbitrary due to variability in enzyme-linked immunoassay antibody cross reactivity (25). However, its use here as a research tool allows binary segregation of subjects into distinct groups with altered postprandial glucose homeostasis for comparison. An individual was considered to have IR if indicated by the OGTT median category of the triplicate assessment, i.e., if two of the three determinations were IR, and then the participant was assigned as IR.

### Postprandial Insulin Response Patterns

Mixed macronutrient tolerance test-dependent insulin response patterns were estimated based on secondary analysis of data reported for 75-g OGTT analyses with 0, 0.5, 1, 2, 3, and 4 h blood collections [30–32]. The following rules established the patterns based on the 0.5, and 3 blood collections in this study. Pattern I – normal = Peak insulin at 0.5 h, 3 h insulin <20% of 0.5 h insulin; Pattern II – delayed insulin decline = Peak insulin at 0.5 h, 3 h insulin <between 20 and 65% of 0.5 h insulin; Pattern III – delayed peak insulin = 3 h insulin >65% of 0.5 h insulin; Pattern IV – high-fasting insulin – 0 h insulin >50 μ Units/ml; Pattern V - low insulin = no insulin >15 μ Units/ml. The low-insulin cut-off was set at 50% of that suggested by Kraft et al. based on the 50% lower 0.5 h insulin in the MMTT vs. OGTT in the iMAPs cohort.

### Fasting Triglyceridemia Assessments

The clinical practice guidelines of the Endocrine Society consider fasting TGs >150 mg/dL and <500 mg/dL as a clinical indication of mild-to-moderate hypertriglyceridemia, posing a risk for cardiovascular disease (26). Therefore, herein, the cutoffs were normal triglyceridemia (fasting TGs <150 mg/dL) or mild-to-moderate hypertriglyceridemia (fasting TGs 150–500 mg/dL) based on these criteria.

### Postprandial Triglyceridemia Assessments

Postprandial triglyceridemia was estimated from areas under the curves of plasma TGs (AUC_TG_) by the trapezoidal rule using either the 0 and 3 h (3h AUC_TG_), or the 0, 3 and 6h (6 h AUC_TG_) TG measurements in mg/dL using the following equations, with results expressed in mg/ml h^–1^:

$$\begin{array}{c} 3\text{h}\;\text{AU}{\text{C}}_{\text{TG}}=\left([\{\text{T}{\text{G}}_{0\text{h}}+\text{T}{\text{G}}_{3\text{h}}/2]\times 3\text{h}\right)/100\\ 6\text{h}\;\text{AU}{\text{C}}_{\text{TG}}=\left(\left[\left\{\text{T}{\text{G}}_{0\text{h}}+\text{T}{\text{G}}_{3\text{h}}\right\}/2\right]\times 3\text{h}\right)+\left(\left[\left\{\text{T}{\text{G}}_{3\text{h}}+\text{T}{\text{G}}_{6\text{h}}\right\}/2\right]\times 3\text{h}]\right)\}/100 \end{array}$$

### Postprandial Triglyceride Kinetic Response Phenotypes

To evaluate phenotypic variability in the rates of plasma TG change in the early and late postprandial periods, available pre-intervention iMAPS MMTT TG data (*n* = 43) were combined with the 340 MMTT-compliant phenotyping participants for a final cohort size of 383 individuals. The rate of TG change in the early period (k_EP_; i.e., 0 to 3 h) and the late period (k_LP_; i.e., 3 to 6 h) were calculated using the zero-order kinetic equations below, with results expressed in mg/dL min^–1^:

$$\begin{array}{c} {\text{k}}_{\text{EP}}=\left[\left(\text{T}{\text{G}}_{3\text{h}}-\text{T}{\text{G}}_{0\text{h}}\right)/180\;\text{min}\right]\\ {\text{k}}_{\text{LP}}=\left[\left(\text{T}{\text{G}}_{6\text{h}}-\text{T}{\text{G}}_{3\text{h}}\right)/180\;\text{min}\right] \end{array}$$

These rates were evaluated in relation to both the 6h AUC_TG_, and the 6 h incremental (i.e., the baseline corrected) area under the postprandial TG curve (incAUC_TG_). The subjects were further stratified into 5 intensity categories of either their 6h AUC_TG_ or incAUC_TG_, using 20% cuts of the population-wide Log (AUC_TG_ + 1) or Log (incAUC_TG_ + 1) ranges.

### Statistics

Data normality was assessed using the Anderson-Darling test, and, if necessary, transformations were optimized to obtain normal distributions prior to effect testing or variable inclusion in modeling efforts. Missing estimates of MMTT intake (*n* = 6 of 491) were replaced with the average intake. Sparse missing data for glucose, insulin, and TG measures were imputed using multivariate normal imputation, considering the entire dataset. Pearson’s correlations were used to evaluate all single-value correlations. Regressions of ISI_Composite_ and ISI_MMTT_ allowed transformation of the ISI_MMTT_ into ISI_Composite_ values. Triglyceridemia classification cutoffs were generated by logistic regression and receiver operator characteristic curve analyses (27). A least squares regression model with ISI_MMTT_ as the outcome variable and BMI, age, sex as the fixed effects with BMI x age and BMI x sex interactions was used to evaluate relationships between these terms. Time-dependent changes in triglyceride levels within identified kinetic groups were evaluated using least squares regression mixed models with plasma triglyceride levels or fasting-corrected triglyceride levels as the outcome variables with time, the triglyceride kinetic pattern group, and AUC_TG_ or incAUC_TG_ intensity groups as fixed effects, with the participant as a random effect, followed by Tukey’s HSD *post hoc* testing. Stepwise linear regressions used to identify factors associated with postprandial phenotypes were used as a decrease in the Bayesian Information Criterion as the stopping function. All statistical analyses were conducted in Jmp Pro v 16 (SAS Institute, Cary NC, United States).

## Results

### Participant Characteristics

The physiological and general health status markers for the study participants are shown in Table 2. While phenotypically more homogeneous, the range of HOMA-IR observed in the iMAPS cohort was similar to that of the phenotyping study subjects and included both insulin-sensitive and insulin-resistant participants.

**TABLE 2:** Participant population baseline blood chemistry, body composition, and metabolic rates.

	Study	iMAPS_Wk1_ (*n* = 43)		Phenotyping (*n* = 340)			
	Sex	Female		Female		Male	
HOMA-IR	Status	Sensitive (*n* = 14)	Resistant (*n* = 29)	Sensitive (*n* = 106)	Resistant (*n* = 66)	Sensitive (*n* = 116)	Resistant (*n* = 52)
Age	y	49 ± 9	49 ± 12	41 ± 13	40 ± 14	40 ± 14	40 ± 14
**Fasting blood chemistry**							
HOMA-IR		1.26 ± 0.48	4.56 ± 2.43^‡^	1.31 ± 0.42	3.53 ± 1.60 ^‡^	1.25 ± 0.42	4.54 ± 4.58^‡^
Glucose	mg/dL	95.3 ± 13.0	102 ± 10	90.4 ± 7.4	99.5 ± 29.0^‡^	93.6 ± 6.9	102 ± 12^‡^
Insulin	mIU/mL	5.5 ± 2.6	18.1 ± 9.3^‡^	5.89 ± 1.80	14.7 ± 6.1^‡^	5.44 ± 1.8	17.4 ± 14.0^‡^
TGs	mg/dL	116 ± 61	138 ± 92	78.5 ± 29.0	119 ± 61^‡^	88.3 ± 36.0	127 ± 60^‡^
NEFA	mEq/L	0.45 ± 0.20	0.38 ± 0.14	0.33 ± 0.15	0.38 ± 0.14^†^	0.27 ± 0.11	0.33 ± 0.14^†^
Total-Chol	mg/dL	203 ± 32	219 ± 33	177 ± 36	181 ± 39	169 ± 32	179 ± 35^†^
LDL-Chol	mg/dL	119 ± 27	139 ± 27^†^	106 ± 30	115 ± 34	108 ± 29	120 ± 33^†^
HDL-Chol	mg/dL	54.7 ± 11.0	49.5 ± 11.0	65.3 ± 17.0	51.9 ± 14.0^‡^	52.6 ± 12.0	42.7 ± 11.0^‡^
**Anthropometric factors**							
BMI	kg/m^2^	31.0 ± 2.7	33.1 ± 4.2	25.5 ± 4	30.5 ± 4.6^‡^	25.3 ± 3.1	31.3 ± 5.2^‡^
Body mass	kg	82.5 ± 11.0	89.5 ± 15.0	67.0 ± 13.0	81.2 ± 14.0^‡^	78.9 ± 12.0	97.9 ± 21.0^‡^
Total fat	kg	35.0 ± 6.2	39.4 ± 7.8	23.1 ± 8.8	33.3 ± 8.7^‡^	16.6 ± 6.5	29.9 ± 13.0^‡^
Lean mass	kg	45.1 ± 5.8	47.9 ± 7.9	41.3 ± 7.0	45.6 ± 74^‡^	59.5 ± 9.1	65.1 ± 9.9^‡^
And. fat	kg	2.95 ± 0.76.0	3.63 ± 1.00^†^	1.62 ± 0.85	2.79 ± 1.00^‡^	1.42 ± 0.77	3.17 ± 1.80^‡^
% Body fat	%	42 ± 3	44 ± 4^‡^	33 ± 8	41 ± 6^‡^	21 ± 7	29 ± 7
And:Gyn		0.49 ± 0.11	0.56 ± 0.13^†^	0.36 ± 0.11	0.48 ± 0.12^‡^	0.47 ± 0.140	0.65 ± 0.18^‡^
Waist Circ	cm	91.1 ± 7.7	98.3 ± 9.9	77.3 ± 9.4	89.4 ± 11.0^‡^	83.9 ± 8.0	100 ± 15^‡^
Waist:Hip		0.80 ± 0.06	0.83 ± 0.06	0.76 ± 0.05	0.80 ± 0.07^‡^	0.85 ± 0.05	0.92 ± 0.08^‡^
**Indirect calorimetry**							
RER_t0_		0.88 ± 0.05	0.85 ± 0.05	0.86 ± 0.05	0.88 ± 0.05	0.86 ± 0.04	0.86 ± 0.05
RER_t0.75–1h_		0.91 ± 0.05	0.88 ± 0.06	0.91 ± 0.05	0.92 ± 0.05	0.90 ± 0.05	0.89 ± 0.04
RER_t3h_		0.89 ± 0.06	0.86 ± 0.04	0.88 ± 0.05	0.89 ± 0.05	0.87 ± 0.05	0.87 ± 0.04
RER_t6h_		0.81 ± 0.04	0.81 ± 0.05	0.82 ± 0.04	0.82 ± 0.04	0.81 ± 0.04	0.82 ± 0.04
REE	kcal/min	1.04 ± 0.15	1.06 ± 0.15	0.987 ± 0.16	1.13 ± 0.18^‡^	1.20 ± 0.18	1.39 ± 0.25^‡^
PPEE_t0.75–1h_	kcal/min	1.15 ± 0.16	1.18 ± 0.15	1.10 ± 0.16	1.22 ± 0.19^†^	1.32 ± 0.19	1.46 ± 0.24^‡^
PPEE_t3h_	kcal/min	1.19 ± 0.12	1.16 ± 0.19	1.11 ± 0.15	1.18 ± 0.18^‡^	1.33 ± 0.19	1.44 ± 0.24^‡^
PPEE_t6h_	kcal/min	1.2 ± 0.12	1.15 ± 0.14	1.07 ± 0.15	1.14 ± 0.19^‡^	1.29 ± 0.18	1.36 ± 0.23^†^

*Results are means ± stdev. Means differences between insulin-sensitive and insulin-resistant groups by sex within each study were assessed after normality transformations and are indicated at <0.05 (†) and <0.001 (‡); HOMA-IR status– HOMA ≥2.05 = insulin resistant; TG – triglycerides; NEFA – non-esterified fatty acids; Chol – cholesterol; And – android fat; Gyn – gynoid fat; RER – respiratory exchange quotient; REE – resting energy expenditure; PPEE – postprandial energy expenditure.*

*ISI_*MMTT*_ calibration with ISI_*Composite*_–* insulin sensitivity assessed by the OGTT-based ISI_Composite_ at the baseline and after 2 and 8 weeks of feeding was unchanged in the iMAPS cohort (16). Therefore, these measures were considered replicate assessments of participant insulin sensitivity. The ISI_Composite_ and ISI_MMTT_ were calculated for each individual and had coefficients of variation (CVs) of 29 ± 19% and 25 ± 14%, respectively. The CVs for HOMA-IR calculated on the OGTT and MMTT test days were similar at 28 ± 24% and 28 ± 17%, respectively. Regression analysis allowed the determination of insulin resistance with the MMTT and a direct comparison of the two calculated ISIs. Based on the experimental data, we transformed the ISI_MMTT_ into the ISI_Composite_ scale using the following equation:

$$\text{Log}\left(\text{IS}{\text{I}}_{\text{Composite}}\right)=\left[0.8751\times \text{Log}\;(\text{IS}{\text{I}}_{\text{MMTT}})\right]-0.2115;\left(r=0.77,\;p<0.001\right)$$

**FIGURE 2:**
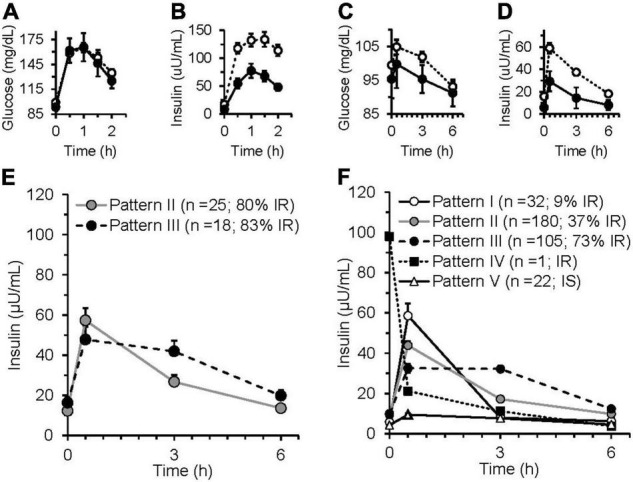
Glucose and insulin responses of insulin-sensitive and insulin-resistant iMAPS participants to a 75 g glucose oral glucose tolerance test (OGTT) and a 50 g sucrose containing mixed macronutrient meal challenge test (MMTT). The OGTT **(A)** glucose and **(B)** insulin responses of participants identified as insulin sensitive (*n* = 8) or insulin sensitive (*n* = 35) by the 2 h ISI_Composite_. The MMTT **(C)** glucose and **(D)** insulin responses of the participants identified as insulin sensitive (*n* = 10) or insulin sensitive (*n* = 33), the 3 h ISI_MMTT_. In panels **(A–D)**: • = insulin sensitive; **○ =** insulin resistance. **(E)** iMAPS participant MMTT-dependent postprandial insulin response patterns. **(F)** Phenotyping participant MMTT-dependent postprandial insulin response patterns. Postprandial response patterns were adapted from previously reported 4 h OGTT-dependent insulin response patterns (29–31). Pattern I – normal = peak insulin at 0.5 h, 3 h insulin <20% of 0.5 h insulin; Pattern II – delayed insulin decline = peak insulin at 0.5 h, 3 h insulin <between 20 and 65% of 0.5 h insulin; Pattern III – delayed peak insulin = 3 h insulin >65% of 0.5 h insulin; Pattern IV – high-fasting insulin – 0 h insulin >50-μ units/ml; Pattern V- low insulin = no insulin >15-μ units/ml. iMAPS results are the mean ± standard error of the means of triplicate measurements at 0, 2, and 8 weeks of intervention. Phenotyping results are means ± standard errors of the participants within each postprandial insulin pattern.

For the iMAPS cohort, the median assignment of the triplicate insulin sensitivity assessments was considered to represent the actual insulin sensitivity status of an individual. The ISI_Composite_ identified 33 individuals with IR (i.e., ISI <4.3), 26 were indicated on all 3 study days. Similarly, the ISI_MMTT_ identified 35 individuals with IR (i.e., ISI <4.3), with 29 on all 3 study days. In all, 39 of 43 (i.e., 90%) of the IR classifications were identical between the two protocols. Of the four that differed, all had borderline assignments. Three individuals were identified with IR on 2 MMTT test days, and 1 OGTT test day, while one individual was identified with IR on 1 MMTT test day, and 2 OGTT test days. By comparison, the HOMA-IR identified 31 individuals as having IR using the sex-specific cutoff of ≥2.05 for women (28). The average postprandial glucose and insulin responses for the iMAPS participants with median insulin-sensitive and insulin-resistant status determined by the OGTT and the MMTT are shown in Figures 2A–D. Five distinct patterns of postprandial insulin responses were identified analogous to those reported by Kraft et al., using OGTTs with 0, 0.5, 1, 2, 3, and 4 h blood draws (29–31). Pattern I represents a normal insulin response, Pattern II indicates a delayed return of postprandial insulin, Pattern III indicates a delayed peak insulin, Pattern IV indicates high-fasting insulin, and Pattern V indicates a low-insulin response. Based on the comparison of the OGTT and MMTT, a low-insulin response to the MMTT was set at 50% of the 30-μU/ml proposed by Kraft et al. As shown in Figure 2E, of the 43 iMAPS women with overweight to obese BMIs, 25 (i.e., ∼60%) showed a delayed insulin decline, and the remainder showed evidence of delayed peak insulin, with both patterns dominated by IR. In the phenotyping participants, all five patterns were detected (Figure 2F). The Insulin-sensitive participants primarily demonstrated Pattern I or V. Of the 123 of 340 individuals (i.e., ∼36%) estimated to have IR, 3 showed insulin Pattern I, while 67 showed insulin Pattern II, and 77 showed insulin Pattern III, while 6% showed Pattern V. A least squares regression model indicated that the Log (ISI_MMTT_) decreased with BMI in both males and females (*p* < 0.001), differing by sex when adjusted for BMI (*p* < 0.001), and while age itself was not a determinant (*p* = 0.2), age-x-BMI interactions indicated that the negative impact of BMI on ISI was elevated in the young (*p* = 0.0003). The prevalence of insulin resistance increased from 21 to 77% in the normal weight, overweight, and obese categories, respectively.

### Triglyceridemia Assessments

Postprandial triglyceridemia following the MMTT was analyzed to assess repeatability of the measurement and to establish cutoffs for hypertriglyceridemia using this tool. The repeated measures in the iMAPS cohort were used to assess the reproducibility of the MMTT TG response, with the median triglyceridemia assignment of the triplicate assessments considered the actual triglyceridemia status for any given individual. The coefficient of variation was 17 ± 10% for the measurement of each time point across the three study visits (t_0h_ = 17 ± 9%; t_0_._5h_ = 17 ± 11; t_3h_ 17 ± 8%, t_6h_ = 16 ± 10%), and 14 ± 7% and 24 ± 16% for the AUC_TG_ and incAUC_TG_, respectively. Therefore, the 6 h MMTT-dependent TG excursions over the 8-week study were reasonably stable. In the phenotyping study, the 6 h postprandial AUC_TG_ calculated using the four available data points was strongly correlated with the 6h AUC_TG,_ not using the 0.5 h data (r^2^ = 1.), the 3h AUC_TG_ (r^2^ = 0.95), and the fasting TG levels (r^2^ = 0.69).

To establish MMTT-based triglyceridemia cutoffs for normal triglyceridemia and mild-to-moderate hypertriglyceridemia, the phenotyping study postprandial TG excursions were compared to fasting triglyceridemia assignments using logistical regression analysis. Specifically, both the 3h and 6h AUC_TGs_ were used to predict the average fasting triglyceridemia assignment in the 340 phenotyping participants, consuming at least 85% of the provided MMTT dose. Postprandial scoring was selected to maximize sensitivity at the expense of 1 specificity of the predicted outcome. As shown in Table 3, fasting TGs established the prevalence of mild-to-moderate triglyceridemia at 11% in the phenotyping participants, while the 3h AUC_TG_ and 6h AUC_TG_ showed 25% and 31 %, respectively. To maximize the mild-to-moderate hypertriglyceridemia identification, the 6h AUC_TG_ was adopted going forward. Applying these estimators to the iMAPS participants, triglyceridemia assignments were generally reproducible across triplicate measures, with inconsistencies being highest in the fasting assessment and increasing as the degree of triglyceridemia approached the cutoffs. Specifically, regardless of the approach used, 37 of 43 (i.e., 86 %) iMAPS study participants showed identical triglyceridemic assignments on all test days. Of the remaining six individuals, fasting TGs indicated a single individual, while the 6h AUC_TG_ indicated three individuals with median assignment of mild-to-moderate hypertriglyceridemia. The 6h AUC_TG_ again estimated a higher degree of mild-to-moderate triglyceridemia (*n* = 22; ∼51%) relative to fasting estimates (*n* = 11; ∼25%) in this group of women with overweight to obese BMIs.

**TABLE 3:** Postprandial and fasting mild-to-moderate hypertriglyceridemia status agreement in the phenotyping study (*n* = 340).

	Fasting TG (mg/mL)	3h AUC_TG_ (mg/mL h^–1^)	6h AUC_TG_ (mg/mL h^–1^)
Cutoff	>1.50	>5.57	>11.15
Mild-Moderate TG	39 (11%)	86 (25%)	105 (31%)
ROC AUC	1.0	0.954	0.939
1-specificity	0	0.166	0.210
Sensitivity	1	1.0	1.0
χ^2^ *p*-value	<0.001	<0.001	<0.001
True Pos/False Pos	39/0	39/47	39/66
True Neg/False Neg	301/0	254/0	235/0

*Fasting triglyceridemia assignment set to “True” in this analysis. AUC_TG_ cutoffs selected to identify all the participants with fasting mild-to-moderate hypertriglyceridemia. AUC_TG_ – area under postprandial triglyceride excursion curve; ROC AUC – area under the receiver operator characteristic curve.*

**FIGURE 3:**
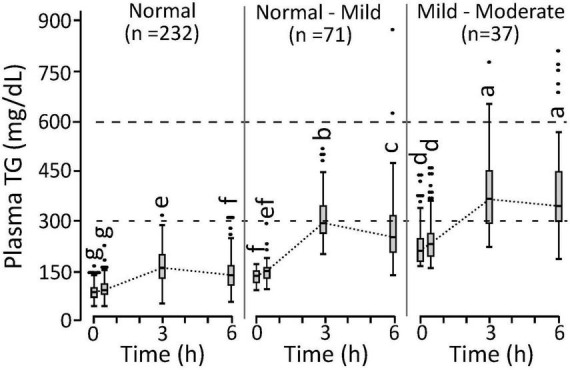
Postprandial triglyceride response in phenotyping cohort individuals characterized with normal triglyceridemia, normal triglyceridemia to mild hypertriglyceridemia, mild-to-moderate hypertriglyceridemia. Cutoffs established for the normal, normal-mild, and mild-moderate postprandial triglyceridemia were <11.15, >11.15 and <14.47, and >14.47 mg/ml h^–1^ for the 6 h area under the triglyceride curve calculated from the 0, 3, and 6 h plasma measurements (i.e., 6h AUC_TG_). Measurements that do not share annotations differ at *p* < 0.05 by Tukey’s HSD *post hoc* analysis. The normal group 0 to 3 h slopes differ from other groups (*p* < 0.0001). The normal-mild group 3 to 6 h slopes differ from other groups (*p* < 0.0016). Results represent means ± standard deviations.

Differences in triglyceridemia assignment between the fasting and postprandial estimators occurred near the assigned cutoffs of each approach. Considering the 71 phenotyping individuals with normal fasting but mild-to-moderate postprandial triglyceridemia, an intermediate normal-to-mild triglyceridemia group with 6h AUC_TG_ between 11.15 and 14.47 mg/dL h^–1^ was established. As shown in Figure 3, this normal-to-mild triglyceridemic group had significantly higher fasting TG levels and a more pronounced postprandial response than the normal triglyceridemia group. Assessing the 383 available participants from the two studies combined, the ranges of measured 6h AUC_TG_ (mg/ml h^–1^) in the normal, normal-to-mild, and mild-to-moderate postprandial triglyceridemia groups were 7.35 ± 1.97 mg/ml h^–1^ (*n* = 249), 14.5 ± 3.4 (*n* = 83), and 19.2 ± 5.8 (*n* = 51), respectively. Moreover, insulin sensitivity decreased as postprandial TGs increased, with the prevalence of IR being 40, 52, and 77% in the normal, normal-to-mild, and mild-to-moderate postprandial triglyceridemia groups, respectively.

### Postprandial Triglyceride Kinetic Response Phenotypes

**FIGURE 4:**
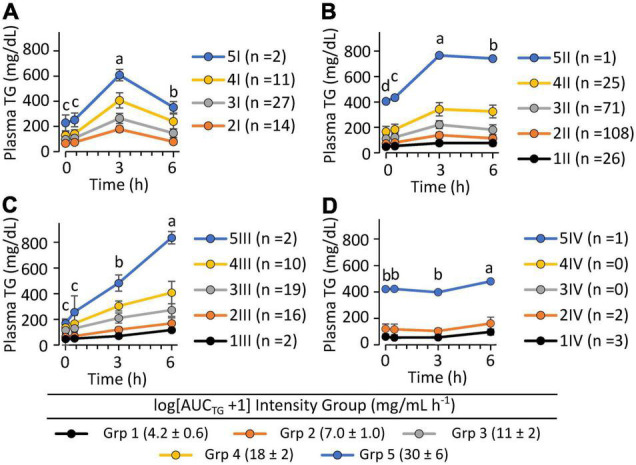
The postprandial triglyceride area under the curve x kinetic response groups among 340 clinically healthy free-living individuals. Data shown are the mean ± SD for the concentrations of the participants identified within five equal intensity groups of the population-wide Log [AUC_TG_ + 1] and one of 4 postprandial kinetic behaviors (Groups I–IV). **(A)** Group I plasma triglycerides appeared quickly and disappeared rapidly (*n* = 54; 16%); **(B)** Group II plasma triglycerides appeared moderately and disappeared slowly (*n* = 231; 68%); **(C)** Group III plasma triglycerides appeared continuously through 6 h (*n* = 49, 14%); **(D)** Group IV plasma triglycerides showed low or delayed postprandial appearance (*n* = 6, 1.8%). Postprandial responses were assigned using the following rules: Group I – [(ka*ke)/AUC_TG_] < 0.033 and [ka/AUC_TG_] >0.021; Group II –0.33 ≤[(ka*ke)/AUC_TG_] < 0.0056 and [ka/AUC_TG_] >0.01; Group III – [(ka*ke)/AUC_TG_] ≥0.0056 and (ka/AUC_TG_) >0.01; Group IV – (ka/AUC_TG_) ≤0.01]. Time-dependent changes in triglyceride levels within identified kinetic groups were evaluated using least squares regression mixed models with plasma triglyceride levels as the outcome variables with time, the AUC_TG_ kinetic pattern group and the AUC_TG_ intensity group as fixed effects, with the participant as a random effect, followed by Tukey’s HSD *post hoc* testing. Time points annotated with different letters within each TG kinetic group are different at *p* < 0.05.

**FIGURE 5:**
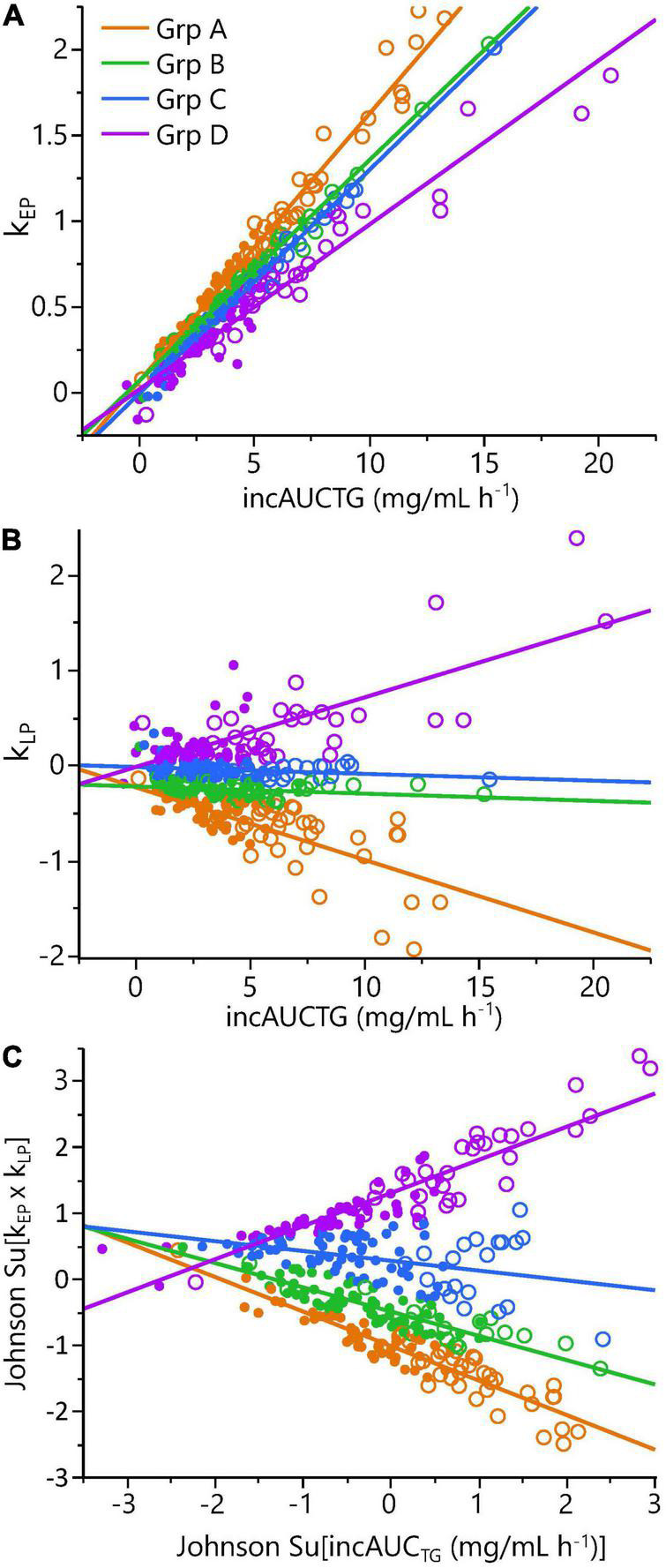
Phenotyping cohort postprandial triglyceride kinetic analysis demonstrated significant phenotypic variance. Panels show: **(A)** the triglyceride rate of change in the 0 to 3 h early phase (k_EP_) as a function of the incremental area under the triglyceride curve (incAUC_TG_); **(B)** the triglyceride rate of change in the 3 to 6 h late phase (k_LP_) as a function of the incAUC_TG_; **(C)** A Johnson-normalized k_EP_ x k_LP_ product as a function of the Johnson-normalized incAUC_TG_. The quartiles of the Johnson Su [(k_EP_ x k_LP_)/incAUC_TG_] defined four kinetic response groups (A-D): Group A – early-phase increase/substantial late-phase decrease (orange; *n* = 86); Group B – early-phase increase/minimal late-phase decrease (blue; *n* = 83); Group C – early-phase increase/no late-phase decrease (green; *n* = 85); Group D – early-phase increase/late-phase increase (purple; *n* = 84). Note, quartiles do not have the same number of participants due to a small percentage of individuals with identical values. The Johnson Su [(k_EP_ x k_LP_)/incAUC_TG_] and k_LP_/incAUC_TG_ differ between each kinetic group by one-way ANOVA with a Tukey *post hoc* analysis (*p* < 0.05). Symbols indicate the estimated postprandial triglyceridemia: • = Normal (6h AUC_TG_ <11.15 mg/ml h^–1^); ○ = mild-moderate (6h AUC_TG_ >11.15 mg/ml h^– 1^).

**FIGURE 6:**
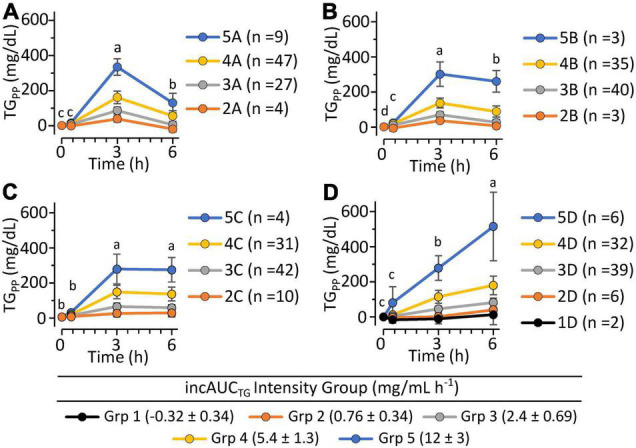
The postprandial triglyceride incremental area under the curve x kinetic response patterns of 340 clinically healthy free-living individuals. Triglyceride kinetic response types A, B, C, and D are defined by the quartiles of the Johnson Su [(k_EP_ x k_LP_)/incAUCTG], which describes the product of the 0 to 3 h and 3 to 6 h triglyceride rates of change in relation to the incremental area under the triglyceride curve. The range of the population-wide Log [incAUC_TG_ + 1] was further subdivided into five equal TG concentration intensity groups. **(A)** Plasma triglyceride kinetic response Group A showed a rapid early period increase and late-period decrease. **(B)** Plasma triglyceride kinetic response Group B showed modest early-period increase with minimal but significant late-period decrease. **(C)** Plasma triglyceride kinetic response Group C showed apparent modest early-period increase but insignificant TG change in the late period. **(D)** Plasma triglyceride kinetic response Group D showed elevating TG levels in both the early and late periods. The occurrence of mild-to-moderate triglyceridemia by the kinetic group was A (*n* = 38; 44%), B (*n* = 19; 22%), C (*n* = 20; 23%) and D (*n* = 30; 36%). Results are means ± stdev of each intensity group. Time-dependent changes in triglyceride levels within identified kinetic groups were evaluated using least squares regression mixed models with fasting-corrected plasma triglyceride levels as the outcome variables, time, the incAUC_TG_ kinetic pattern group, and the incAUC_TG_ intensity group as fixed effects, and the participant as a random effect, followed by Tukey’s HSD *post hoc* testing. Time points annotated with different letters within each TG kinetic group are different at *p* < 0.05.

To evaluate phenotypic variability, the TG k_EP,_ and k_LP_, the 43 complete pre-intervention iMAPS study MMTT dataset was combined with the 340 MMTT-compliant phenotyping study dataset, yielding a cohort of 383 individuals. As shown in Figure 4, despite equivalent intake, considerable variability in postprandial serum TG behavior was observed. Grouping subjects based on their TG k_EP_ and k_LP_ as a function of the AUC_TG_ defined four kinetic pattern groups: Group I - TG increase until 3 h and decreased to 6 h (*n* = 54; 16%); Group II – TG increased until 3 h and changed little between 3 and 6 h (*n* = 231; 68%); Group III – TG increased continuously through 6 h (*n* = 49, 14%); Group IV - no change in TG between 0 and 3 h, and a minimal increase at 6 h (*n* = 6, 2%). To characterize the TG concentration range distribution by the TG kinetic group, the population-wide AUC_TG_ was also subdivided into 5 intensity categories of equal ranges [i.e., 20% cuts of the observed Log (AUC_TG_ + 1) range]. Notably, the rates of TG change before and after the 3 h time point were independent of the fasting TG concentration. While k_EP_ and k_LP_ are linked, failure to obtain a return to baseline levels for most participants, not to mention the distribution of TGs between various lipoprotein particles with their own kinetic behaviors, prevents a true assessment of the meal TG absorption and elimination rates. However, as shown in Figure 5, the product of the k_EP_ and k_LP_ (i.e., k_EP_ x k_LP_), when combined with the incAUC_TG,_ provides a useful phenotypic descriptor of the postprandial TG behavior, with the quartiles of the Johnson normalized [(k_EP_ x k_LP_)/incAUC_TG_], establishing four TG kinetic pattern groups, denoted A, B, C, and D. The distribution of the five incAUC_TG_ intensity groups [i.e., 20% cuts of the observed Log (incAUC_TG_ + 1) range] across these four incAUC_TG_ kinetic groups is shown in Figure 6. While k_LP_ was negatively correlated with k_EP_ (*p* < 0.0001), considerable k_EP_-independent variability was observed. Translating this phenotypically, we propose that, after TGs appeared, they could disappear quickly, slowly, or continue to rise at the 6 h postprandial measurement. While the AUC_TG_ intensity categories were distributed across all incAUC_TG_ kinetic patterns, Group A had higher prevalence of mild-to-moderate hypertriglyceridemia than other kinetic phenotypes (A = 44%; B = 22%; C = 23%; D = 36%; χ^2^ *p* < 0.01).

### Stability of Mixed Macronutrient Tolerance Test Triglyceride Kinetic Response

**FIGURE 7:**
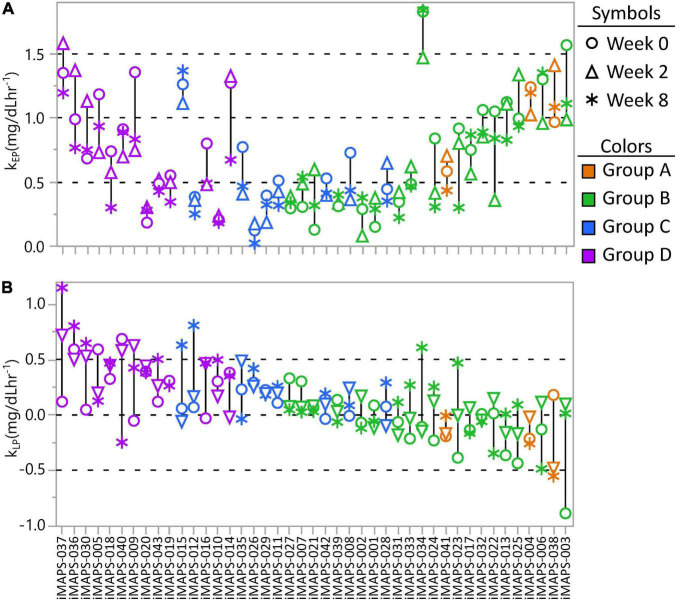
The intra-individual variance in **(A)** early-phase postprandial triglyceride change (k_EP_) and **(B)** late-phase postprandial triglyceride change (k_LP_) from the plasma measured at 0, 2, and 8 weeks of the iMAPS dietary intervention. The 43 participants are ordered by decreasing k_EP_ x k_LP_ and colored by the median k_EP_ x k_LP_ kinetic quartile group (A, B, C, or D) of the three measurements. Considering all participants, and controlling for the participant as a random effect, _EP_ increased (*p* = 0.012) and k_LP_ tended to decrease (*p* = 0.06) over the 8-week intervention, but was not affected by diet type, age, or BMI.

To assess the stability of the estimated TG k_EP_ and k_LP_ in responses to the MMTT, the kinetic group quartile cutoffs from the 383 measurements were applied to the 2-week and 8-week postprandial TG results of the iMAPS participants. As with the ISI and triglyceridemia assessments, the median categorical Johnson Su [(k_EP_ x k_LP_)/incAUC_TG_] phenotype assignment of the 0-, 2-, and 8-week measurements was used to indicate the “true” phenotype of each participant. Substantial variability was observed in both the k_EP_ and k_LP_ (Figure 7) as well as their products. Considering the [(k_EP_ x k_LP_)/incAUC_TG_], a 39 % misclassification rate was observed between the weekly and median kinetic group classification. Of the 43 participants, 6 (14 %) showed all measures in a single (k_EP_ x k_LP_) group, and 16 (37%) had all measures assigned to one of the two adjacent groups (e.g., A-B-A). However, 12 participants (28 %) had at least two measures in one group, but a third of at least two groups away (e.g., A-A-D) and nine (21%) were assigned to different groups at each visit. The overall k_EP_CV was 30 ± 18%, and, for rates >0.5 mg/dL min^–1^ (*n* = 26), CVs were 26 ± 13%. In contrast, the overall k_LP_ CV was 300 ± 765%, and, for rates >0.2 mg/dL min^–1^ (*n* = 18), CVs were 64 ± 38 %. Moreover, when controlling for the participant as a random effect, the 8-week dietary intervention was associated with a weak but significant decrease in k_EP_ (*p* = 0.0038) and increase of k_LP_ (*p* = 0.025). This relationship was reflected in strong correlations between the incAUC_TG_ and k_EP_ at 2 and 8 weeks, with the 0-week data (r^2^ ∼0.6, *p* < 0.001), but weaker relationships in k_LP_; (2-week vs. 0-week and 8-week k_LP_- r^2^ ∼0.2, *p* < 0.01; 0-week vs. 8-week k_LP_- *p* > 0.05). Therefore, the elimination rate appears to introduce the greatest degree of variability into this assessment of postprandial TG kinetic behavior.

### Postprandial Triglyceride Kinetic Associations With Body Fat Distribution and Energetics

**FIGURE 8:**
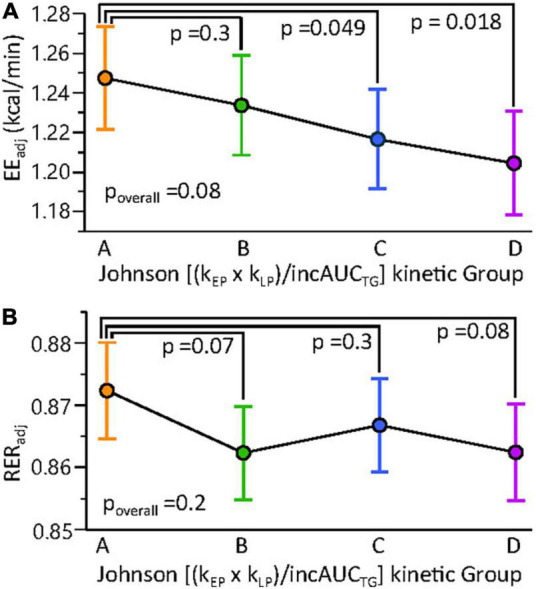
Resting and postprandial energy metabolism shows subtle differences among MMTT triglyceride kinetic response groups. Mixed models of **(A)** resting and postprandial energy expenditure (EE) and **(B)** the respiratory exchange ratio (RER) were constructed using lean body mass, BMI, time, and the Johnson (k_EP_ x k_LP_)/incAUC_TG_ kinetic quartile group as fixed effects and the participant as random effect. Results are adjusted least square means ± standard errors. Results of contrast post-tests of Group A vs. other kinetic groups are shown. Constructing the same models with Johnson (k_EP_ x k_LP_)/incAUC_TG_ as a continuous variable indicated a negative correlation between this factor and EE (*p* = 0.0056) but not RER (*p* = 0.1).

To identify factors associated with the rate of TG change in the early and late postprandial periods, associations between the k_EP_ and k_LP_ with physiological and metabolic factors were assessed. To simplify kinetic evaluations, the six subjects without minimal postprandial TG elevations (Figure 4) were removed from this analysis. Fasting TGs were strongly correlated with k_EP_ (*n* = 375; RMSE = 0.92; r^2^ = 0.25; *p* < 0.0001). In stepwise linear regressions, considering body composition parameters, a model of k_EP_ showing positive associations with the log of the android to the gynoid fat ratio [Log (And:Gyn); *p* < 0.0001] and negative associations with body mass (*p* = 0.0095) described ∼9% of the variance in this parameter (*n* = 375; RMSE = 0.96; r^2^ = 0.085; *p* < 0.0001). Including indirect calorimetry measures in the stepwise linear regression, k_EP_ was best predicted by a model, including positive correlations with the Log [And:Gyn] and the baseline respiratory exchange ratio (RER_t0_; *n* = 375; RMSE = 0.86; r^2^ = 0.16; *p* < 0.0001). Similar results were found for AUC_TG_ and incAUC_TG_ (data not shown). In contrast, a linear model of k_LP_ explained ∼5% of this factor (*n* = 375; RMSE = 0.39; r^2^ = 0.06; *p* < 0.0002) and included negative associations with the postprandial energy expenditure at 3 h (EE_3h_; *p* = 0.0001), the 0.75-1 h postprandial RER (RER_0.75–1h_; *p* = 0.016) and an interaction between these components (*p* = 0.037). We then ran a mixed model regression of EE, including the lean body mass, BMI, postprandial time, and either Johnson Su [(k_EP_ x k_LP_)/incAUC_TG_] or the Johnson Su [(k_EP_ x k_LP_)/incAUC_TG_]-defined kinetic group as main effects, with the participant as a random effect (Figure 8). The covariate-adjusted EE was negatively correlated with the Johnson Su [(k_EP_ x k_LP_)/incAUC_TG_] (*p* = 0.00056), with Group A having higher EE than Groups C and D. Similar results were observed with the postprandial V̇O_2_ and V̇CO_2_, both of which were negatively correlated with k_LP_ after adjusting for time, lean body mass, and BMI (*p* < 0.0005). While RER was negatively correlated with k_LP_ when adjusted by time and lean body mass (*p* = 0.038), significant differences by the kinetic group were not observed.

## Discussion

The metabolic dysregulation of glucose and TG homeostasis is linked to multiple adverse health outcomes, including type 2 diabetes, cardiovascular disease, stroke, and cognitive impairment (32). While these risk factors are associated with being overweight, variable risk and resilience to these metabolic perturbations exist in the general population and across BMI categories (33–35). Today, the assessments of glycated hemoglobin (HbA1c), fasting glucose, TGs, and cholesterol are commonly used to detect early signs of metabolic dysfunction and metabolic disease progression. If fasting serum glucose suggests the presence of type 2 diabetes, an oral OGTT may be prescribed to confirm diagnosis. Less appreciated is pre-diabetes, which is estimated to be present in ∼30% of the U.S. population. Lipemic risks are typically estimated from fasting plasma TGs and cholesterol levels (26, 36). While total cholesterol levels are minimally influenced by the postprandial state, remnant cholesterol [i.e., total cholesterol minus both high-density lipoprotein cholesterol (HDLc) and low-density lipoprotein cholesterol (LDLc)] is strongly correlated with postprandial triglycerides (37). Moreover, elevations in hepatic export and/or reductions in peripheral TG-rich lipoprotein clearance following a meal can result in prolonged hypertriglyceridemia despite normal fasting TG levels (38). Regardless of whether remnant cholesterol, triglycerides, or both are linked to disease risk and progression, non-fasting TG levels are clinically relevant cardiometabolic risk factors, independent of fasting TGs, LDLc, and HDLc (3, 36). Thus, while fasting blood analyte concentrations are useful for diagnosing cardiometabolic disease, probing the non-fasted state has clinical value and may aid in the identification of pre-emergent disease or differential disease risk across the population. While the clinical assessment of glucose tolerance is routine, standardized assessments of lipid tolerance are still being refined (14, 36, 39, 40). Mixed meal tests induce robust metabolic multi-organ responses that collectively reflect the adaptive responses to metabolic challenges, with limited metabolic flexibility indicating poor metabolic health (41, 42). While MMTT recommendations exist, the macronutrient sources and balance of reported MMTTs are quite variable, with broad ranges of fat (22–80 g), carbohydrate (11–75 g), and protein (3–36 g) being reported (36, 39, 42–47). In the current study, we evaluated a liquid MMTT, delivering ∼59-g palm oil, ∼59-g sucrose, and ∼29-g egg white protein to perturb both insulin and TG homeostasis. From our results in over 380 individuals, we harmonized cutoffs for postprandial insulin sensitivity and hypertriglyceridemia with reported cut-points using OGTTs and meal challenge tests, while evaluating the intra- and inter-individual variability in responses.

Previous studies have demonstrated that mixed meal challenges can accurately assess insulin sensitivity (9, 42, 44). Our results concur with these findings and extend them by allowing their transformation into an OGTT frame of reference, allowing seamless integration with the historical literature. Assessing insulin sensitivity by both a standard 2 h OGTT and the 6 h MMTT in a parallel crossover design provided nearly equivalent IR determinations and with similar precision. Specifically, for triplicate measures conducted over an 8-week intervention period, any single OGTT or MMTT measurement showed an ∼85% chance of identifying the median OGTT-assessed insulin sensitivity status for an individual. Moreover, inconsistencies in each test were highest for the participants with borderline values. It was also clear that, despite the lack of a 2 h and 4 h postprandial blood draw, sufficient resolution existed in the sampling design to identify phenotypic variation in postprandial insulin patterns that have been associated with the detection of occult diabetes (29–31).

Ingestion of a lipid-rich meal can be used to probe postprandial lipid handling, allowing segregation of individuals based on both their basal and dynamic lipid metabolism. Oral fat tolerance tests (OFTTs) are valuably clinically relevant tools for investigating postprandial lipid metabolism (14, 36, 39). Classically, OFTTs are conducted over 6 h with hourly sampling (39, 48). Reducing the OFTT to a 4 h duration can provide reliable postprandial lipemia assessments in most subjects but is less reliable in hypertriglyceridemic individuals (45, 47). Considering the ∼1.6 h lag in oral TG absorption (49), shorter time points have been deemed inappropriate. The current study supports this conjecture, as a significant proportion of the population had 6 h plasma TG levels above their 3 h time point, resulting in hypertriglyceridemia indications in more individuals. These findings suggest that a large segment of the population has plasma TG enrichment at these late postprandial time points, as previously reported with smaller cohorts (50). Optimizing postprandial TG cutoffs to identify fasting hypertriglyceridemic subjects (i.e., >150 mg/dL as defined by the Endocrine Society), we established both 3h and 6h AUC_TG_ cutoffs, demarking normal, normal-to-mild, and mild-to-moderate hypertriglyceridemia. When applied to the 340 phenotyping participants, the 3 h and 6 h measures showed 37% and 6% misclassification rates compared to the 200-mg/dL cutoff for any post-OFTT TG measurement suggested by an expert panel of scientists and clinicians (36). Therefore, a 6h AUC_TG_ cutoff of 11.15 mg/ml h^–1^, following the MMTT defined here, appears clinically relevant. However, while fasting TG measures indicated only 11% mild-to-moderate hypertriglyceridemia, the 6h AUCTG increased this to 31%. Moreover, ∼20% of the individuals showed 11.15 to 14.47 mg/ml h^–1^ of TGs, defining an intermediate group with apparently normal triglyceridemia to mild hypertriglyceridemia. Future studies should evaluate the cardiometabolic risk associated with postprandial TGs in this region relative to that >14.47 mg/ml h^–1^ of TGs, the apparent threshold for mild-to-moderate hypertriglyceridemia.

While the AUC_TG_ integrates postprandial triglyceridemia into a single manageable value, the incAUC_TG_ provides a better measure of the postprandial response to a high-fat meal (51). Regardless, both approaches mask the underlying kinetic behavior that can be used to phenotypically stratify subjects and provide insight into physiological mechanisms, driving postprandial lipemia (48, 52). It has become clear that post-ingestion, an early phase TG release from an enterocyte storage pool, occurs prior to the primary postprandial peak commonly occurring between 3 and 4 h (48). Using basic pharmacokinetic principles, the postprandial behavior can be segregated into pseudo-absorptive and pseudo-elimination phases, corresponding to the measurable appearance and disappearance of TGs in the blood stream. In the current study, we used the 0 to 3 h and 3 to 6 h periods to demark these early and late phases and found an array of patterns in both the magnitude and temporal kinetics of individual responses. While the average overall response and that of most subjects showed the highest measured TG concentration at the 3 h time point, roughly half of those individuals plateaued at that level and a quarter continued to rise through 6 h. Similarly, the patients with treatment-resistant cardiovascular disease and normal fasting TGs ingestion of a high-fat meal resulted in TG maximum at 4 h, where they plateaued and remained above fasting levels for up to 12 h after a high-fat meal (53). Another study compared the 6 h postprandial response to a high-fat test meal in normolipidemic lean and obese individuals, and mildly obese hyperlipidemic subjects with <20 individuals per group (52). In that study, obesity and fasting TG status were found to increase the timing of peak triglyceridemia, as well as the maximum concentration obtained, with substantial variability within groups. In the current study, while BMI and total body fat were not correlated with k_EP_, the And:Gyn ratio adjusted by total body mass showed a weak positive association with this rate. We also found that the k_EP_ was positively correlated with fasting TG levels and higher in the hyperlipidemic than normolipidemic subjects. Previous studies have reported such associations between fasting and postprandial TG responses (39, 54, 55). Interestingly, k_EP_ was also positively correlated with the RER_t0_, suggesting that the rates of triglyceride increases were higher when participant baseline fat oxidation rates were lower. Moreover, upon adjusting for RER, the And:Gyn ratio was a positive predictor of k_EP_ regardless of sex, again consistent with other reported links between fat depot distribution and postprandial triglyceridemic responses (56). The TG k_LP_ was considerably more variable, showing influences by both the magnitude of TGs achieved and postprandial whole-body energy metabolism. Specifically, results suggest that, when controlling for lean body mass, time, BMI, and the k_EP_, EE increased as the late phase kinetic rate decreased. The inverse relationship between the EE and k_LP_ may reflect either subtle differences in fuel availability or utilization. Regardless, such inter- and intra-individual variability in TG clearance is not surprising, considering the complex physiological dynamics between the intestine, liver, muscle, and adipose tissue, along with genetic influences, that control postprandial TG levels in the plasma (39, 48). The infrequent sampling implemented here likely further contributes to the high variance in k_LP_. If plasma TG levels continued to rise after 3 h as expected in hypertriglyceridemic individuals (45, 47, 53), substantial error in the TG clearance estimate would be inherent in the collected data. Regardless, individuals with 6 h TGs that exceed their 3 h levels (i.e., those with a “D-response” type in our study) will have prolonged and underappreciated postprandial lipemia. In particular, AUC_TG_-based determinations of postprandial hypertriglyceridemia in such individuals would tend to undercount this condition, since those with borderline levels may exceed the 200 mg/dL at times after 6 h. Finally, if cardiometabolic risk is associated with the time that an individual maintains plasma TGs above a particular threshold, one would expect those with a “D-response type” would be at higher risk, as daily intake from multiple meals would be expected to exacerbate plasma TGs to a larger extent in these individuals. As a first step, future studies evaluating the multiple meal effects in individuals with these MMTT-defined postprandial TG phenotypes appear warranted.

## Limitations

It should be appreciated that all postprandial indices of insulin sensitivity are influenced by other physiological factors, including beta-cell function and glucose absorption rates and must be interpreted carefully (22, 57). While the MMTT pretest dinner was controlled in both the iMAPS and phenotyping studies, these meals differed considerably with respect to the macronutrient balance when comparing the two studies. However, both dinners were relatively high carbohydrate meals relative to the MMTT. It has been reported that such a precursor high carbohydrate meal can blunt fat catabolism and may, therefore, exaggerate some assessments of postprandial triglyceridemia in morning meal challenges (48). Furthermore, a relatively small group of female participants (*n* = 43) were used to calibrate the MI_MMTT_ cutoffs, and sex-specific cutoffs for HOMA-IR have been reported. In addition, sex-dependent differences in lipid metabolism are known, and the identified triglyceridemic cutoffs should not be considered an indication differential risk of cardiometabolic disease (58, 59). The lack of a 2 h blood draw does pose some difficulties in distinguishing between Pattern II and Pattern II postprandial responses, but the method is equivalent to an OGT for assessing insulin sensitivity. Finally, the limited number of postprandial blood draws likely increased the variability of postprandial TG kinetic assessments, particularly in the late postprandial phase. Therefore, measurements appearing to increase from 3 to 6 h may have plateaued prior to the terminal blood draw. Regardless, these individuals would appear to have prolonged postprandial triglyceridemia.

## Conclusion

Using 0, 3, and 6 h blood draws following the ingestion of an MMTT comprised of a 840-kcal palm oil (60 cal%), sucrose (28 cal%), and egg white protein (12 cal%) liquid meal allowed for the simultaneous determination of insulin sensitivity and postprandial triglycerideimia status in clinically healthy individuals. The MMTT was acceptable to 95% of the participants. The 3 h ISI_MMTT_ was transformed into a 75 g OGTT ISI_Composite_ frame of reference and provided an equivalent indication of IR, with a cutoff of <4.3. This tool identified ∼36% of individuals in the phenotyping cohort with some impairment in carbohydrate metabolism, with prevalence increasing with BMI. Due to the recruitment strategy, this should not be interpreted as the prevalence of IR in the population. While IR was detected in individuals with normal, overweight, and obese BMIs, it became more prevalent at higher android fat distributions. As compared to fasting triglyceridemia assessments, a 6 h MMTT AUC_TG_ of >11.15 mg/ml h^–1^increased the detection of hypertriglyceridemia from 11 to 31% in the phenotyping cohort. Moreover, this cut point provided equivalent stratification of normal triglyceridemia and mild-to-moderate hypertriglyceridemia indicated by any MMTT postprandial TG >200 mg/dL. Interestingly, ∼25% of the population showed rising plasma TGs through 6 h after intake, with rates of TG disappearance being weakly associated with the ability to metabolize fats. Therefore, the described procedures using an MMTT prepared from commonly available food materials provide results equivalent to an OGTT and OFTT in a single test, reporting on perturbations in both glucose homeostasis and daylong triglyceridemia.

## Data Availability Statement

Requests for raw data supporting the conclusions of this article should be made by email to the corresponding author. Requests will be reviewed quarterly by a committee consisting of the study investigators.

## Ethics Statement

The studies involving human participants were reviewed and approved by University of California, Davis Institutional Review Board. The patients/participants provided their written informed consent to participate in this study.

## Author Contributions

JN, SA, CS, and NK conceived and developed the research plan. JN, SK, NK, and CS conducted the research. JN performed the statistical analysis. SK and KB provided statistical review. JN wrote the primary manuscript. SK, KB, NK, and SA provided significant editorial input of manuscript. All authors have primary responsibility for final content and read and approved the final manuscript.

## Conflict of Interest

The authors declare that the research was conducted in the absence of any commercial or financial relationships that could be construed as a potential conflict of interest.

## Publisher’s Note

All claims expressed in this article are solely those of the authors and do not necessarily represent those of their affiliated organizations, or those of the publisher, the editors and the reviewers. Any product that may be evaluated in this article, or claim that may be made by its manufacturer, is not guaranteed or endorsed by the publisher.

## Abbreviations

And: Gyn – android to gynoid fat ratio
AUC: area under the curve
BMI: body mass index
DXA: dual-energy x-ray absorptiometry
EE: energy expenditure
HDLc: high-density lipoprotein cholesterol
HOMA-IR: homeostasis model of insulin resistance
iMPAS: individual metabolism and physiological signatures study
incAUC: incremental area under the curve
IR: insulin resistance
ISI: insulin sensitivity index
k_EP_: rate of change in plasma triglycerides between 0 and 3 h
k_LP_: rate of change in plasma triglyceride between 3 and 6 h
LDLc: low-density lipoprotein cholesterol
MMTT: mixed macronutrient tolerance test
OFTT: oral fat tolerance test
OGTT: oral glucose tolerance test
RER: respiratory exchange ratio
TGs: triglycerides.
