# Supplementary material for: Assessing Insulin Sensitivity and Postprandial Triglyceridemic Response Phenotypes With a Mixed Macronutrient Tolerance Test
Source: Front Nutr. 2022 May 11;9:877696. doi: 10.3389/fnut.2022.877696 (PMC9131925; doi:10.3389/fnut.2022.877696)
Supplement: Supplementary file 1 [file Data_Sheet_1.PDF]

Pre-Test Meal Components

FL95 iMAPS

Gram	Food	Brand
100.0	Tortilla, Flour	Mi Ranchito
65.7	Chicken Patty	Sysco Classic
20.0	FL95 Rice	See recipe
23.2	Black Beans	Casa Solano
25.0	Cheese Sauce	See recipe
46.4	Refried Beans	See recipe
10.0	Cheddar Cheese	Casa Solano
30.0	Corn Chips	La Tapatia
185.0	Lemonade	Minute Maid

FL100 Phenotyping

Gram	Food	Brand
100.0	FL100 Rice	See recipe
80.0	Scrambled Egg	Grand Prairie
80.0	Mixed Vegetables	Sysco Imperial
30.0	Corn	C&W
90.0	Sweet and Sour Sauce	Sysco Imperial
1.2	Sesame Seeds	Sysco Imperial
226.0	White Bean-Potato Soup	See recipe
150.0	Raspberry Sorbet	Edy's/Dreyer's
185.0	Lemonade	Minute Maid

Pre-Test Meal Recipes

FL95 Rice

%	INGREDIENTS	BRAND	VENDOR
21.95%	Rice, Brown, Parboiled	PAREXCL	SYSCO
7.08%	Onion, Yellow, Diced	PACKER	SYSCO
48.67%	Stock, Chicken, Unsalted	SWANSON	CAMPBELL'S
3.54%	Oil, Olive, Extra Virgin	CA OLIVE RANCH	CA OLIVE RANCH
8.96%	Juice, Tomato, Low Sodium	CAMPBELL'S	CAMPBELL'S
0.18%	Cumin, Ground	MCCORMICK	SAFEWAY
0.78%	Garlic Paste	GARDEN GOURMET	SAFEWAY
8.85%	Salsa, Garden-Pepper (Red)	PACE	CAMPBELL'S

PROCEDURE

1. Set oven to 350F.
2. Combine stock, juice, cumin, garlic paste, and salsa. Whisk to dissolve garlic paste.
3. Add oil and onions to large stockpot. Sauté over medium heat for 3 min.
4. Add rice, and stir with long-handled heat-resistant spatula for 3 more minutes.
5. Add stock mixture, and stir thoroughly. (Be careful to not get burned by the steam).
6. Remove from heat, and carefully transfer to a shallow hotel pan. Cover with a solid lid.
7. Carefully transfer to preheated oven; and bake for 60 minutes.
8. Carefully remove from oven and cool on cooling rack for 15 minutes.
9. Fluff, and then transfer to WIR to marry and chill overnight.
10. Portion and freeze.

Back to top

Cheese Sauce

%	INGREDIENTS	BRAND	VENDOR
67.42%	Milk, Evaporated, Nonfat	CARNATION	SAFEWAY
1.69%	Thickening Solution	See recipe	See recipe
9.11%	Product, Cheese, Velveeta	VELVEETA	SYSCO
12.54%	Cheese, Mozzarella, Shredded	AREZIMP	SYSCO
9.11%	Cheese, Cheddar, Full Fat	CASASOL	SYSCO
0.05%	Turmeric, Ground	MCCORMICK	SAFEWAY
0.05%	Paprika, Hungarian, Ground	SZEGELD	SAFEWAY
0.02%	Gum, Xanthan	BOB'S RED MILL	BOB'S RED MILL

PROCEDURE

1. Heat milk and thickening solution in large stockpot over medium-low heat to simmer, stirring constantly (be sure to scrape bottom while stirring).
2. When the milk has thickened, remove from heat. Add the Velveeta, and stir until mostly melted.
3. Stir in the mozzarella and cheddar cheeses until mostly melted.
4. If needed, return to a very low heat, stir constantly until the cheese is melted and sauce is smooth.
5. Sprinkle in the turmeric, paprika, and xanthan gum; whisk until thoroughly incorporated.
6. Transfer to shallow hotel pans and chill overnight.
7. Use in other recipes: corn chowder, southwest chowder, and enchiladas. Then portion remaining.

Back to top

Refried Beans

%	INGREDIENTS	BRAND	VENDOR
69.09%	Water, Deionized	MK&HFL	SYSCO
30.91%	Beans, Refried, Dehydrated	CASASOL	SYSCO

PROCEDURE

1. Bring 2 quarts of water to a boil in a 4-quart pot.
2. Place beans in a shallow hotel pan.
3. Pour the boiling water over the beans. Stir briefly.
4. Cover and let stand for 5 minutes.
5. Stir once more, and then transfer to walk-in refrigerator.
6. Chill overnight. Portion and freeze.

Back to top

FL100 Rice

%	INGREDIENTS	BRAND	VENDOR
15.28%	Rice, Brown, Parboiled	PAREXCL	SYSCO
15.28%	Rice, White, Parboiled	PAREXCL	SYSCO
69.44%	Stock, Vegetable, Organic	O-ORGANICS	SAFEWAY

PROCEDURE

1 Preheat oven to 350F.
2 Weigh rice in shallow hotel pan.
3 Add stock to weight in hotel pan with rice.
4 Stir rice immediately following the addition of stock.
5 Bake, covered, for 60 minutes.
6 After rice is cooked, set aside on cooling rack for 15 minutes.
7 Transfer to walk-in refrigerator to chill overnight.
8 Portion and freeze.

Back to top

White Bean-Potato Soup

%	INGREDIENTS	BRAND	VENDOR
54.95%	Stock, Vegetable	O-ORGANICS	SAFEWAY
0.003%	Coloring, Caramel	DEAN	SYSCO
0.08%	Mushrooms, Boletes, Dried	INTLSUP	SYSCO
1.95%	Margarine, Soy-Free	EARTH BALANCE	SAFEWAY
8.11%	Onion, Diced	PACKER	SYSCO
4.87%	Celery, roughly sliced	SAFEWAY	SAFEWAY
0.11%	Sugar, Granulated	C&H	SYSCO
0.53%	Garlic Paste	GARDEN GOURMET	SAFEWAY
0.16%	Ginger Paste	GARDEN GOURMET	SAFEWAY
12.68%	Beans, White, Drained, Rinsed	SYSCO CLASSIC	SYSCO
0.49%	Consommé Powder, Kosher, Meat-Free	OSEM	SAFEWAY
1.10%	Thickening Solution	See recipe	See recipe
0.03%	Gum, Xanthan	BOB'S RED MILL	BOB'S RED MILL
1.95%	Kale, Frozen, Chopped	EARTHBOUND FARMS	SAFEWAY
12.98%	FL100 Roasted Potatoes	See recipe	See recipe
0.02%	Pepper, White, Ground	MCCORMICK	SAFEWAY

PROCEDURES

1 Combine stock, caramel coloring, and mushrooms into one large plastic pitchers. Set aside.
2 Add margarine, onion, celery, and sugar into a large stock pot.
3 Sauté on setting 1, stirring frequently until thoroughly caramelized (~25 minutes).
4 Turn off heat, and stir in garlic and ginger thoroughly.
5 Add vegetable stock solution, scraping the bottom of the stock pot to capture caramelized bits.

6 Add beans & consommé powder. Set heat to 2. Bring to boil; stir frequently to avoid scorching.
7 Add thickening solution, and stir until thickened. Remove from heat, and add xanthan gum.
8 Let sit for 10 minutes, then carefully transfer to two large Waring Blenders. Lock the lids.
9 Purée on low in the large Waring blender for 1 minute. Repeat with other batch.
10 Return purée to a rinsed stockpot, and add the potatoes and kale.
11 Set heat to 1. Bring to a boil, stirring constantly to avoid scorching.
12 Turn off heat and add the white pepper.
13 Transfer to shallow hotel pans, and refrigerate overnight according to procedures.

Back to top

Thickening Solution

%	INGREDIENTS	BRAND	VENDOR
57.48%	Water, Deionized	WHNRC	WHNRC
21.13%	Starch, Modified Food	AMERICAN SPICE	AMERICAN SPICE
9.04%	Starch, Arrowroot	BOB'S RED MILL	BOB'S RED MILL
12.17%	Starch, Tapioca	BOB'S RED MILL	BOB'S RED MILL
0.17%	Gum, Xanthan	BOB'S RED MILL	BOB'S RED MILL

PROCEDURE

1 Weigh ingredients separately.
2 Carefully transfer dry ingredients into the bowl of the food processor.
3 With the motor running, steadily pour the water through the hole in the lid until comes together.
4 Open lid, and using a hard scraper, scrape down sides and bottom of bowl to loosen chunks.
5 Use a rubber spatula to further scrape residue into solution.
6 Turn on motor again, and process until homogenized.
7 Transfer to squeeze bottle.

Back to top

FL100 Roasted Potatoes

%	INGREDIENTS	BRAND	VENDOR
98.45%	Potatoes, Red-Skinned, Diced, Frozen	LAMB	SYSCO
1.55%	Oil, Canola	SYSCO CLASSIC	SYSCO

PROCEDURE

1 Set racks in Wolf convection ovens to the 2,4,6,8,10 positions.
2 Set dial on Wolf convection oven to 325, with FAN SET TO HIGH.
3 Line 8 full-sized sheet pans with two pieces of parchment paper each.
4 In the large stockpot, combine the potatoes and the oil.
5 Using two large rubber spatulas, toss potatoes to evenly coat with oil.

6 Transfer potatoes to parchment-lined sheet pans.
7 Bake for 20 minutes, then carefully mix the potatoes with a heat-stabile spatula.
8 Bake for and additional 10 minutes.
9 Place sheet pans on a short bun rack, and roll into walk-in freezer. Set timer for 2 hours.
10 After 2 hours, transfer to large 4-mL plastic bags. Label and freeze until needed.

Back to top
